# Glutamatergic Activation of Neuronostatin Neurons in the Periventricular Nucleus of the Hypothalamus

**DOI:** 10.3390/brainsci10040217

**Published:** 2020-04-06

**Authors:** Sema Serter Kocoglu, Duygu Gok Yurtseven, Cihan Cakir, Zehra Minbay, Ozhan Eyigor

**Affiliations:** 1 Department of Histology and Embryology, Balikesir University School of Medicine, Balikesir 10145, Turkey; 2 Department of Histology and Embryology, Sanko University School of Medicine, Gaziantep 27090, Turkey; dgok@uludag.edu.tr; 3 Department of Histology and Embryology, Bursa Uludag University School of Medicine, Bursa 16240, Turkey; cihancakir10@gmail.com (C.C.); zminbay@uludag.edu.tr (Z.M.); oeyigor@uludag.edu.tr (O.E.)

**Keywords:** neuronostatin, Glutamate, c-Fos

## Abstract

Neuronostatin, a newly identified anorexigenic peptide, is present in the central nervous system. We tested the hypothesis that neuronostatin neurons are activated by feeding as a peripheral factor and that the glutamatergic system has regulatory influences on neuronostatin neurons. The first set of experiments analyzed the activation of neuronostatin neurons by refeeding as a physiological stimulus and the effectiveness of the glutamatergic system on this physiological stimulation. The subjects were randomly divided into three groups: the fasting group, refeeding group, and 6-cyano-7-nitroquinoxaline-2,3-dione (CNQX)+refeeding group. We found that refeeding increased the phosphorylated signal transducers and transcription activator-5 (pSTAT5) expression in neuronostatin-positive neurons and that the CNQX injection significantly suppressed the number of pSTAT5-expressing neuronostatin neurons. The second set of experiments analyzed the activation pathways of neuronostatin neurons and the regulating effects of the glutamatergic system on neuronostatin neurons. The animals received intraperitoneal injections of glutamate receptor agonists (kainic acid, α-amino-3-hydroxy-5methyl-4-isoazepropionic acid (AMPA), and N-methyl-D-aspartate (NMDA)) or 0.9% NaCl. The number of c-Fos-expressing neuronostatin neurons significantly increased following the AMPA and NMDA injections. In conclusion, we found that the neuronostatin neurons were activated by peripheral or central signals, including food intake and/or glutamatergic innervation, and that the glutamate receptors played an important role in this activation.

## 1. Introduction

Neuronostatin (NST), encoded by the somatostatin gene, is an anorexigenic peptide with 16 amino acids [1]. Immunohistochemical studies have shown that the bodies of neuronostatin-positive neurons are prominent in the hypothalamic anterior periventricular and suprachiasmatic nuclei (SCN), while those of neuronostatin-positive axon terminations are prominent in the arcuate nucleus and median eminence. Also, there are fewer and less densely-marked neuronostatin-expressing cells in the polymorphic layer of the dentate gyrus and motor cortex, amygdala, and cerebellum [2]. The presence of neuronostatin neurons and neuronostatin-positive axon terminations in the areas of the hypothalamus that control food intake, the demonstration of decreased water and food intake in animals following intraventricular neuronostatin administration, and the decreasing dose-dependent and short-term food intake in mice after intraperitoneal neuronostatin administration all suggest that neuronostatin may play a role in the control of appetite and metabolism [3,4,5]. 

In the central nervous system, glutamate is the major excitatory amino acid neurotransmitter and its importance in the regulation of the neuroendocrine systems and the hypothalamus-pituitary-endocrine system axis is well documented [6,7]. Glutamate-mediated neurotransmission occurs via G-protein-mediated metabotropic and ion channel-forming ionotropic glutamate receptors. These receptors are classified according to their agonists: N-methyl-D-aspartate (NMDA), α-amino-3-hydroxy-5methyl-4-isoazepropionic acid (AMPA), and 2-carboxy-3-carboxymethyl-4-isopropenylpyrrolidine (Kainate) receptors [8].

One of the methods used to demonstrate glutamatergic innervation in target neurons is to label some specific molecules involved in neurotransmission with different techniques. Glutamate synthesized in the soma is transported to the axon terminals through vesicular glutamate transporters (VGLUT). VGLUTs are molecules specific to the glutamatergic system, and they are used as specific markers of neurons that use glutamate as neurotransmitters in immunohistochemical techniques [9]. However, immunohistochemically, they can only be shown in the terminal boutons of the axon [10]. Therefore, the presence of VGLUTs on the boutons of the target neuron indicates that the neuron receives glutamatergic innervation [11]. In addition, the demonstration of glutamate receptor subunit proteins that mediate the glutamate effect in the target neuron is another indicator of glutamatergic innervation. However, the presence of receptors does not give information about the functionality of the receptors. Therefore, the response of the target neuron to glutamate should be evaluated. For this purpose, animals are administered only glutamate receptor subunit agonists or antagonists/agonists peripherally or centrally, and then changes in the expression of neuronal activation markers in the neuron are determined using immunohistochemical methods.

Transcription factors involved in the regulation of gene expression are specific proteins that play an active role in neuronal functioning, neurogenesis, and neuronal innervation. The transcription factors can be activated in the different intracellular signal transduction pathways, or they can be directly activated by ligands, such as glucocorticoids and some vitamins [12,13,14]. Certain transcription factors are activated by phosphorylation. These transcription factors are phosphorylated in the cytoplasm and translocate to the nucleus to initiate transcription. Immunohistochemically, the expression of proteins in the nucleus is used as a marker of neuronal activation. Transcription factors, such as c-Fos, phosphorylated cAMP response element-binding protein (pCREB), or phosphorylated signal transducers and transcription activators (pSTATs) have been used as markers for determining neuronal activity changes [15,16,17,18].

Despite the many publications describing the mechanisms of action of neuronostatin, there is no data about the peripheral and central control systems that play a role in the regulation of neuronostatin neuron activation. Therefore, we hypothesized that neuronostatin neurons are activated by feeding as a peripheral factor and the glutamatergic system has regulatory influences on neuronostatin neurons. The first set of experiments analyzed the activation of neuronostatin neurons by refeeding as a physiological stimulus and the effectiveness of the glutamatergic system on this physiological stimulation. The second set of experiments analyzed the regulating effects of the glutamatergic system on neuronostatin neurons.

## 2. Materials and Methods

### 2.1. Animals

All animal experiments were performed according to the National Institute of Health Guide for the Care and Use of Laboratory Animals. The experimental procedures were approved by the Experimental Ethical Committee of Bursa Uludag University (Approval No: 2016–4/4). 60-day-old male Sprague-Dawley rats (200–250 g) (*n* = 45, male), obtained from the Bursa Uludag University Laboratory Animal Breeding, Usage and Research Center, were used in this study. Animals were housed in this center where the light cycle and the temperature were controlled (a 12:12 h light–dark cycle with the lights off at 7:00 am at 21 °C) with freely available water).

### 2.2. Experimental Groups

Experiment 1: Investigation of the effect of refeeding as a physiological stimulus on neuronostatin neurons and the effectiveness of the glutamatergic system.

For this purpose, three experimental groups were designed consisting of male rats, with *n* = 5 per group. After a 48-h fasting period; the refeeding group was allowed to eat ad libitum for 2 h, while the fasting group was unfed and the antagonist group was injected intraperitoneally with non-NMDA glutamate antagonist 6-cyano-7-nitroquinoxaline-2,3-dione (CNQX) 15 min before the 2-h refeeding period (2 mg/kg CNQX in 300 µL distilled water). After the 48-h fasting, refeeding was started at 9:00 am at the beginning of the dark period of the dark–light cycle and the animals were allowed to feed for 2 h. The CNQX injections were performed at 9:00 am. 

Experiment 2: Investigation of the regulating effects of the glutamatergic system on neuronostatin neurons.

Six groups were set up to determine the effect of glutamate on neuronostatin neurons (*n* = 5 per group). The first group was designated as the kainic acid group. The rats in this group were intraperitoneally injected with kainic acid (2.5 mg/kg in 300 µL distilled water, DW). The kainic acid control group received saline injections (300 µL saline, intraperitoneal, ip). AMPA was administrated to the rats of the second experimental group (AMPA group, 5 mg/kg in 750 µL DW, ip). The AMPA control group received 750 µL of saline (ip). The third group of rats, the NMDA group, was given NMDA (100 mg/kg in 2 mL DW), and the NMDA control group was injected with 2 mL saline (ip). 

All drugs were administered between 9:00 a.m. and 11:00 a.m. Ninety minutes after injection, deep anesthesia was performed using ether, and the animals were sacrificed by transcardial perfusion with 4% paraformaldehyde (PFA) in 0.13 M Sorenson’s phosphate buffer, pH 7.4 (300 mL/animal). All brains were removed and post-fixed at +4 °C overnight in 4% paraformaldehyde. Five series of brain sections with a thickness of 40 μm were cut using a vibratome. The sections along the rostral-caudal axis of the hypothalamus were collected in Tris-HCl buffer (0.05 M, pH 7.6). The brain sections were then washed in buffer and stored at −20 °C in cryoprotectant until use.

### 2.3. Immunohistochemistry

Between incubations, we washed the sections in Tris-HCl three times. A blocking buffer was used to dilute the primary and the secondary antibodies. Blocking buffer was prepared in Tris-HCl buffer containing normal horse serum (10%), Triton X-100 (0.2%), and sodium azide (0.1%). Before the primary antibody incubation, the sections were exposed to blocking buffer for 2 h for the prevention of non-specific binding. 

The sections were brought to room temperature and the cryoprotectant was removed by buffer washes. Free floating sections were then incubated in preheated antigen retrieval (AR) solution (final solution temperature 73–75 °C) for 30 min. Trisodium citrate buffer (50 mM, pH 6, for neuronostatin) or ethylenediaminetetraacetic acid (EDTA) solution (1 mM, pH 8, for phosphorylated signal transducers and transcription activator-5 (pSTAT5)) was used in the AR process. Following the AR, endogenous peroxidase activity was stopped using 3% H_2_O_2_ and then the sections were treated with blocking buffer. Primary antibody incubation for c-Fos (rabbit anti-c-Fos, 1/20,000, Chemicon, Billerica, MA, USA) was carried out overnight at room temperature. 

For pSTAT5 immunohistochemistry, rabbit anti-pSTAT5 antibody (1/2000 dilution, 9351, Cell Signaling Technology, Danvers, MA, USA) was used for 6 nights at +4 °C. After the primary antibody incubation, the sections were transferred into the secondary antibody solution containing biotin conjugated donkey anti-rabbit immunoglobulin G (IgG, 1/300, Jackson Immunoresearch Labs, West Grove, PA, USA) and incubated for 2 h. The sections were then washed three times and exposed to avidin-biotin complex (ABC Elite Standard Kit, Vector Labs, Burlingame, CA, USA). Diaminobenzidine (DAB) solution (25 mg DAB, 2 g nickel ammonium sulfate, 2.5 µL hydrogen peroxide in 100 ml Tris-HCl buffer) was used in order to make the immunoreactive signal visible under the microscope. 

For the second primary antibody detection of the double immunohistochemistry, the sections were blocked and incubated in rabbit anti-neuronostatin antibody (1/4000 dilution, H-060-50, Phoenix Pharmaceuticals, Inc., Burlingame, CA, USA) for three nights at room temperature. A secondary antibody (biotin conjugated donkey anti-rabbit IgG (1/400, Jackson Immunoresearch Labs, West Grove, PA, USA) incubation step was performed for 2 h after the primary antibody incubation. Sections were then exposed to a avidin-biotin complex and DAB solution (25 mg DAB, 2.5 µL hydrogen peroxide in 50 mL Tris-HCl buffer). At the end of the double-immunostaining, the sections were mounted on slides, dried, and coverslipped with DPX. For the negative control experiments primary antibody or secondary antibody steps were omitted.

### 2.4. Statistical Analysis

Sections were analyzed and the images were captured with an Olympus BX-50 photomicroscope attached to a charge-coupled device (CCD) camera (Olympus DP71, 1.5 million pixels, Olympus Corporation, Tokyo, Japan). Sections between the coordinates (bregma-0.24 mm to −3.60 mm for the periventricular nucleus), determined according to the rat brain atlas [19], were used for single and double immunohistochemical labeling. We used cross sections taken at five different levels at the same coordinate and at equal distance for each animal in the rostrocaudal plane for cell counting. In dual indirect immunoperoxidase-labeled sections, the ratio of c-Fos- or pSTAT5-expressing neurons to all neuronostatin neurons was calculated for each animal. Counts were performed ‘blindly’ and by two different researchers, and all neuronostatin neurons in the periventricular nucleus were counted. The intra-group mean and standard error of the mean (SEM) of the percentages obtained for each subject were determined. The analysis of variance between the experimental groups was statistically compared using a one-way ANOVA test, and *p* < 0.05 was considered significant. 

## 3. Results

### 3.1. Neuronostatin Neuron Immunoreactivity in the Hypothalamus

**Figure 1:**
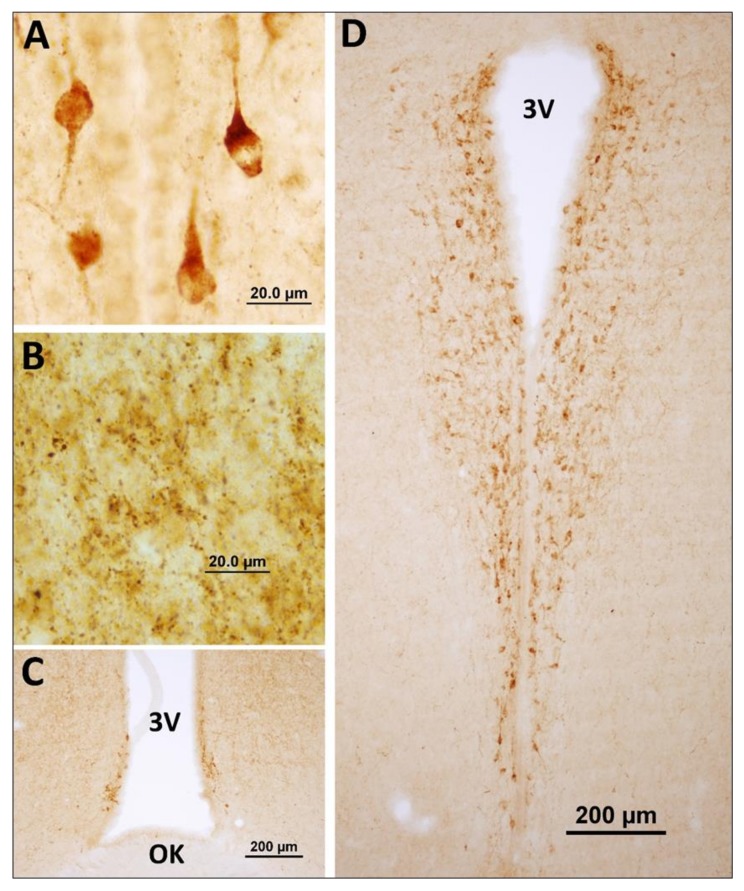
Distribution of neuronostatin neurons in the hypothalamus. (**A**,**C**,**D**) Neuronostatin neurons located around the third ventricle in the periventricular nucleus of the anterior hypothalamus. (**B**) Neuronostatin-positive axonal outcomes observed in ventromedial hypothalamic nucleus (VMH). 3V: third ventricle, OK: optic chiasm.

**Figure 2:**
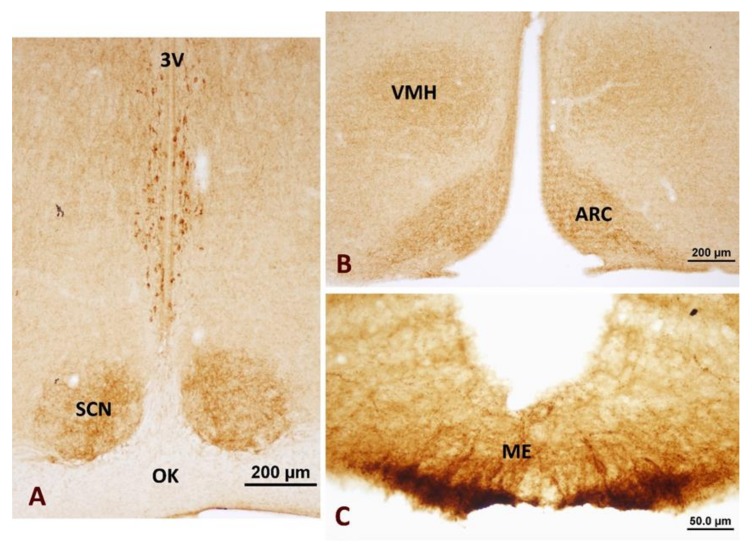
Distribution of neuronostatin-positive axon terminals in the hypothalamus. (**A**) Suprachiasmatic nucleus (SCN), (**B**) ventromedial hypothalamic nucleus (VMH) and arcuate nucleus (ARC), and (**C**) median eminence. 3V: third ventricle, OK: optic chiasm.

Neuronostatin immunoreactive neuron bodies were expressed in the anterior hypothalamic periventricular nucleus around the third ventricle (Figure 1). Neuronostatin-positive axon terminals were localized in the suprachiasmatic nucleus (Figure 2A), ventromedial hypothalamic nucleus with arcuate nucleus (Figure 2B), and median eminence (Figure 2C).

### 3.2. Physiological Stimulation of Neuronostatin Neurons and Investigation of the Effects of Glutamate Antagonists

**Figure 3:**
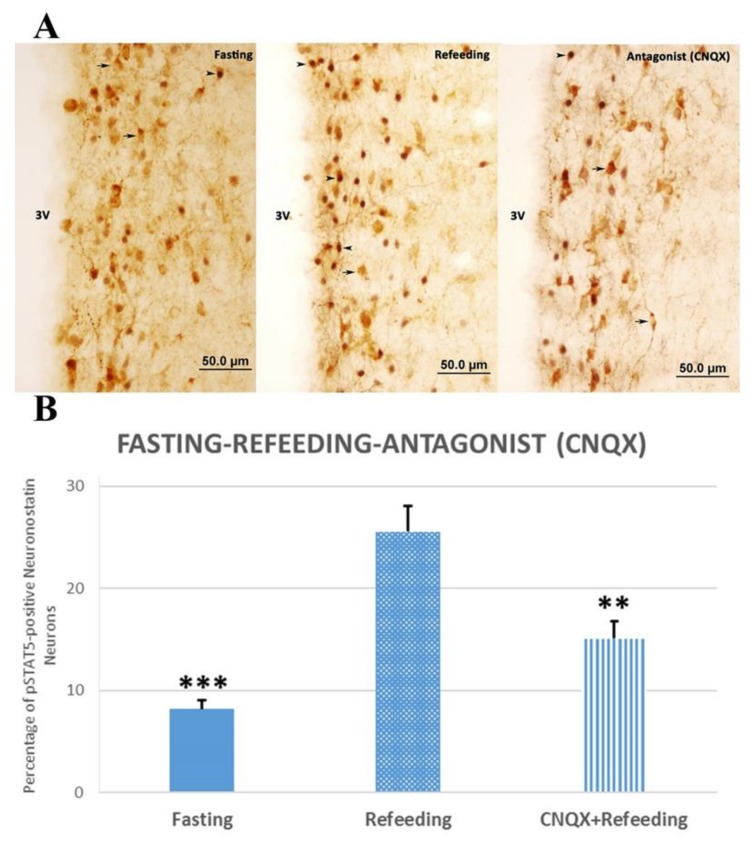
The effect of CNQX, a non- N-methyl-D-aspartate (NMDA) glutamate antagonist, in subjects stimulated by refeeding after fasting. There were significant differences between fasting-refeeding groups (*** *p* < 0.001) and refeeding-antagonist groups (** *p* < 0.01) (**B**). pSTAT5 expression in neuronostatin neurons after 48 h fasting (on the left), pSTAT5 expression in neuronostatin neurons activated with refeeding in anterior hypothalamic periventricular nucleus (in the middle), neuronostatin neurons in the anterior periventricular nucleus in CNQX-treated subjects before feeding (on the right) (**A**). pSTAT5 protein-expressing neuronostatin neurons (▲) and pSTAT5-negative neuronostatin neurons (🡹). 3V: third ventricle.

We found that 2-h refeeding after 48-h fasting induced phosphorylated signal transducers and transcription activator-5 (pSTAT5) expression in neuronostatin neurons of the anterior hypothalamic periventricular nucleus. The 2-h refeeding after 48-h fasting caused a significant increase in the number of pSTAT5-positive neuronostatin neurons when compared with the fasting group (*p* < 0.001, Figure 3). The injection of specific antagonist CNQX prior to agonist significantly decreased the percentage of pSTAT5-positive neuronostatin neurons (*p* < 0.01, Figure 3).

In the refeeding group, about 25.56 ± 2.52% of the neuronostatin neurons localized in the periventricular zone were pSTAT5-positive, whereas this ratio was 8.18 ± 0.87% in the fasting group. The ratio of the activated neuronostatin neurons was reduced to 15.08 ± 1.73% after CNQX injection (Figure 3). 

### 3.3. Investigation of the Glutamatergic System Effects and Activation Pathways in the Regulation of Neuronostatin Neurons

In the anterior hypothalamic periventricular nucleus, c-Fos expression in neuronostatin neurons was detected following the glutamate agonist administration. The greatest increase in the number of c-Fos expressing neuronostatin neurons was caused by the NMDA injection. 

**Figure 4:**
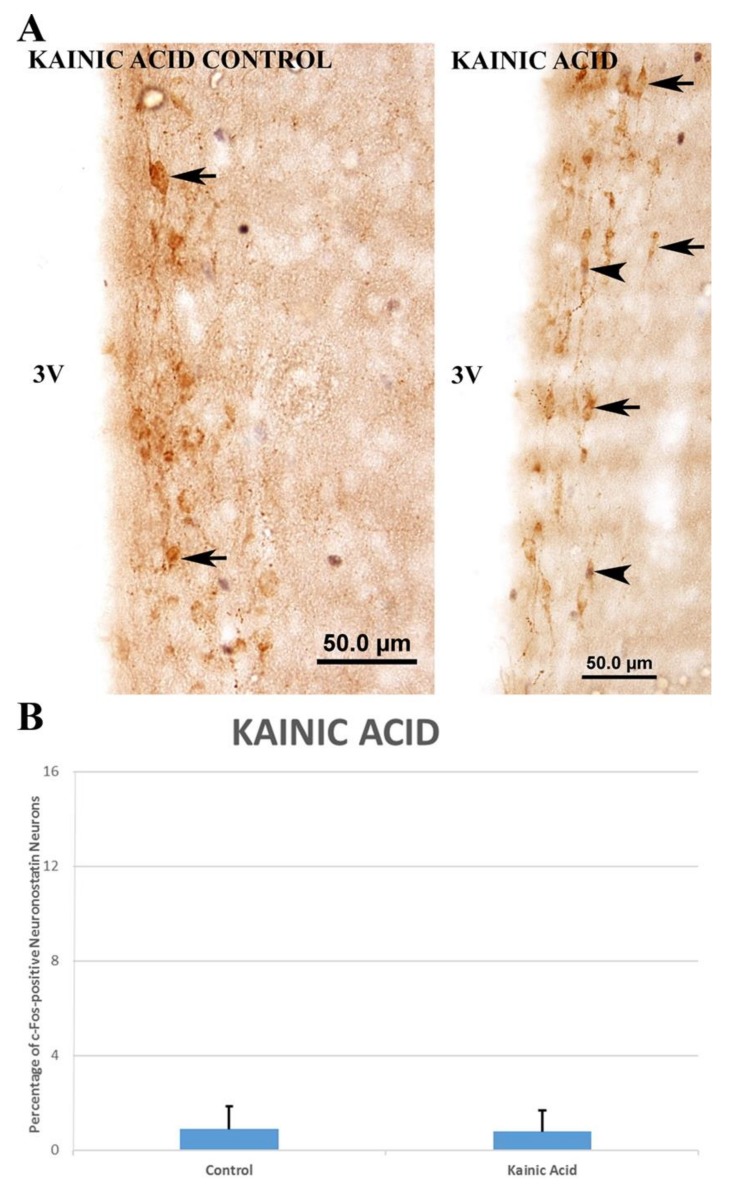
Effect of kainic acid administration on c-Fos expression in neuronostatin neurons located in the anterior hypothalamic periventricular nucleus (**A**). c-Fos protein-expressing neuronostatin neurons (▲) and c-Fos-negative neuronostatin neurons (🡹). No significant differences were found between the control and kainic acid groups (**B**). 3V: third ventricle.

**Figure 5:**
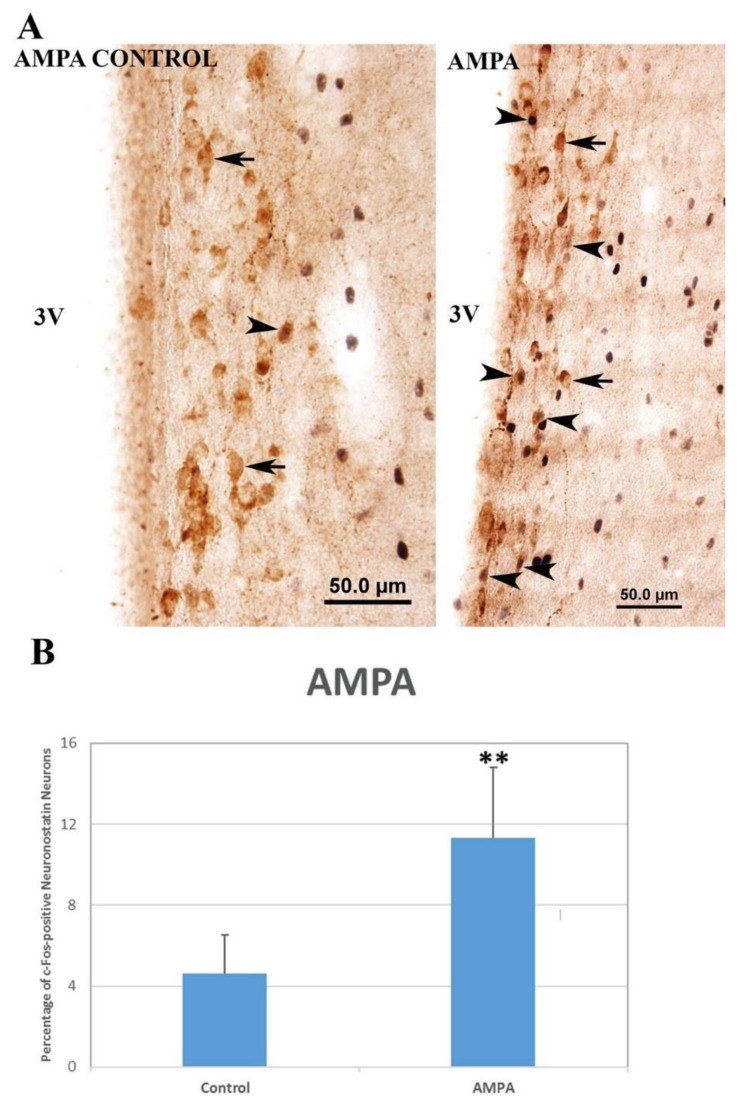
The effect of AMPA administration on c-Fos expression in neuronostatin neurons located in the anterior hypothalamic periventricular nucleus. c-Fos protein-expressing neuronostatin neurons (▲) and c-Fos-negative neuronostatin neurons (🡹). c-Fos expression in neuronostatin neurons localized in the anterior hypothalamic periventricular nucleus (**A**). Significant differences were found between the control and AMPA groups (** *p* < 0.01) (**B**). 3V: third ventricle.

**Figure 6:**
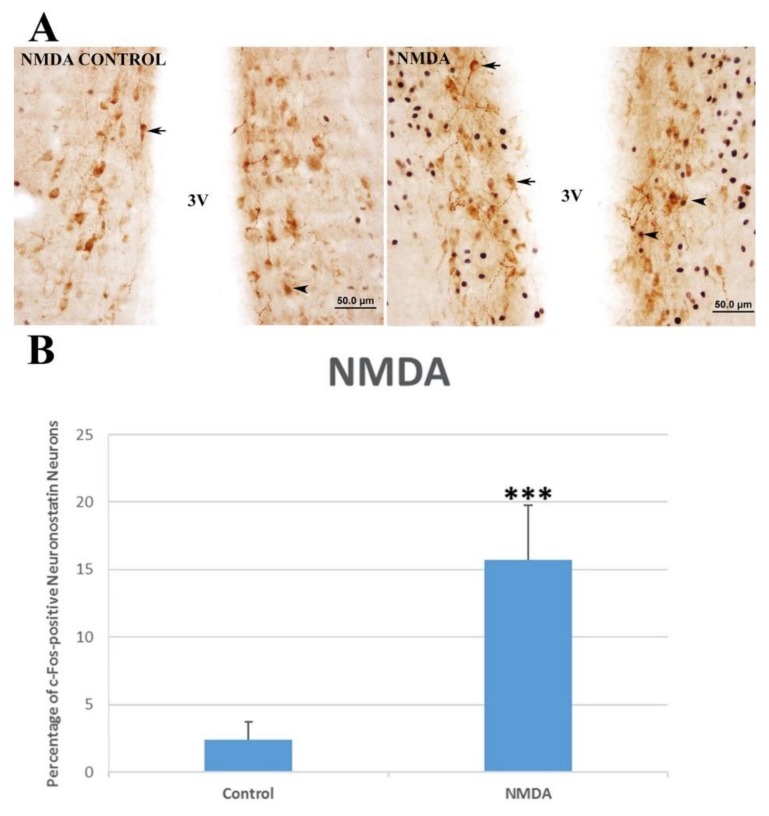
The effect of NMDA administration on c-Fos expression in neuronostatin neurons located in the anterior hypothalamic periventricular nucleus. c-Fos protein-expressing neuronostatin neurons (▲) and c-Fos-negative neuronostatin neurons (🡹). c-Fos expression in neuronostatin neurons localized in the anterior hypothalamic periventricular nucleus (**A**). Significant differences were found between the control and NMDA groups (*** *p* < 0.001) (**B**). 3V: third ventricle.

In the kainic acid group, there was no significant increase (0.78 ± 0.51% vs. 0.88 ± 0.55%) in the number of c-Fos-positive neuronostatin neurons when compared to the control group (Figure 4). The number of c-Fos-positive neuronostatin neurons was significantly increased by AMPA (11.31 ± 1.56% vs. 4.6 ± 0.86%, *p* < 0.01) (Figure 5) and NMDA (15.74 ± 1.80% vs. 2.37 ± 0.58%, *p* < 0.001) (Figure 6) in comparison to the control group. 

## 4. Discussion 

Food intake is under the control of numerous neurotransmitter and neuropeptide systems. The role of hypothalamic peptides in the control of food intake is an intensive area of investigation. Neuronostatin is a newly identified peptide that has regulatory effects on nutrient and water uptake, energy consumption, the cardiovascular and digestive systems, and is expressed by neurons localized in the anterior hypothalamic periventricular nucleus. Although there is some literature on the effects of neuronostatin neurons in the target cells and organs, no experimental studies have been done on the central neurotransmitter neurons (such as neurotransmitters and neuropeptides) and peripheral (food intake) regulators. Therefore, we investigated the effects of the glutamatergic system on neuronostatin neurons, as this system plays a role in the regulation of the activity of many neuroendocrine neurons in the hypothalamus, which uses glutamate as a neurotransmitter of excitatory amino acid with a peripheral factor such as refeeding.

### 4.1. Hypothalamic Distribution of Neuronostatin Neurons

Immunohistochemical studies have shown that neuronostatin-positive neurons are localized in the anterior hypothalamic periventricular nucleus and SCN, while neuronostatin-immunoreactive axon terminations are localized in the arcuate nucleus with median eminence. There are fewer and less densely-marked neuronostatin-expressing cells in the polymorphic layer of the dentate gyrus and motor cortex, amygdala, and cerebellum [2]. Consistent with the knowledge in the literature, the immunohistochemical results of this study showed that the neuronostatin-immunoreactive neuron soma was localized in the anterior hypothalamic periventricular nucleus, while the axon endings were localized in the median eminence, the suprachiasmatic nucleus, the ventromedial hypothalamic nucleus, and the arcuate nucleus. In addition, neuronostatin-positive neurons were not observed in other hypothalamic nuclei. The results of our study suggested that the neuronostatin bodies localized in the anterior hypothalamic periventricular nucleus formed a network via the neuronostatin axons localized in other nuclei of the hypothalamus. Our study suggested that the neuronostatin neurons localized in the anterior hypothalamic nucleus may be associated with the adenohypophysis via the neuronostatin axon endings localized in the median eminence.

### 4.2. Investigation of the Glutamatergic System Effects and Activation Pathways in the Regulation of Neuronostatin Neurons

The regulatory effects related to neurogenesis, apoptosis, neurite development, and synapse formation of glutamate, the main excitatory neurotransmitter of the central nervous system, on the hypothalamic neuroendocrine systems, has been intensively investigated [20,21,22,23,24,25]. In the present study, the ionotropic glutamate agonists (kainic acid, AMPA, and NMDA) were administered to investigate the effects of the glutamatergic system on neuronostatin neurons. In this study, the sub-seizure doses of the agonists were used and no indication of seizure was observed in the animals. 

Reports in the literature have suggested that kainic acid [25,26,27,28], AMPA [29,30], and NMDA [29,30,31,32,33] can cross the blood–brain barrier and reach the central nervous system. Our immunohistochemical studies showed that systemic administration of ionotropic non-NMDA and NMDA glutamate receptor agonists directly or indirectly activate neuronostatin neurons at different rates and that the intracellular pathway of c-Fos protein plays a role in this activation. To our knowledge, this is the first report in the literature describing the stimulation of neuronostatin neurons by any neurotransmitter systems.

In the experimental groups, the presence and rate of activated neuronostatin neurons varied according to the type of agonist. AMPA and NMDA administration increased the number of c-Fos- positive neuronostatin neurons significantly, whereas kainic acid administration did not show any significant difference. In the subjects, the increase in the number of activated neuronostatin neurons was observed to be about seven-fold with agonist NMDA and 2.5-fold with agonist AMPA. These results suggest that glutamate exerted its effect on neuronostatin neurons by forming homomeric and/or heteromeric functional AMPA and NMDA receptors, and not via kainate receptors. This suggests that neuronostatin neurons responded less to non-NMDA receptor agonists than NMDA. In another of our studies, the ratio of c-Fos positive nesfatin neurons was determined in kainic acid, AMPA, and NMDA injected subjects to be 50%, 78%, and 87%, respectively. Statistical evaluation between agonists showed that activation with kainic acid injection was significantly less than with the other two agonists (AMPA and NMDA). In addition, we demonstrated expression of the ionotropic glutamate receptors with fluorescence microscopy on neuronostatin neurons in the periventricular nucleus of the hypothalamus (preliminary unpublished data). 

### 4.3. Physiological Stimulation of Neuronostatin Neurons and Investigation of the Effects of Glutamate Antagonists

Neuronostatin peptides have a suppressive effect on liquid intake as well as food intake [1,34]. Food intake and liquid intake are independent behaviors [35]. While liquid intake continues under physiological conditions without nutrient intake, food intake occurs under conditions in which liquid intake is restricted [36]. In addition, a reduced liquid intake is thought to lead to a decreased food intake [35]. Thus, it is recommended that the two behaviors be separately evaluated when designing experiments that investigate the possible effects of peptides on nutrient and fluid intake [35]. In our study, subjects were allowed access to water up to euthanasia, suggesting that fasting-induced refeeding and/or liquid intake may cause activation in neuronostatin neurons.

STAT5 was first reported as a prolactin-stimulated mammary gland factor [37]. There are studies showing that the STAT5 pathway was activated by feeding-related hormones and cytokines (such as granulocyte-macrophage colony-stimulating factor (GM-CSF)). For example, leptin administration caused STAT5 activity in some hypothalamic nuclei, such as arcuate and supraoptic nuclei, of male subjects [38]. In our study, fasting-induced refeeding in male animals led to the activation of neuronostatin neurons and this activation was mediated by the intracellular pathway with pSTAT5.

This data supports the idea that the STAT5 pathway has a role in regulation of the activities of neuroendocrine neurons. Furthermore, it is plausible that the STAT5 pathway is involved in the physiological activation of neuronostatin neurons when challenged by refeeding after fasting. In our study, 2-h refeeding after 48-h fasting induced the activation of pSTAT5-positive neuronostatin neurons in the anterior hypothalamic periventricular nucleus and this activation was significantly suppressed by glutamate receptor antagonist (CNQX) application. Previous studies suggested that CNQX can reach the central nervous system and affect the neuronal activation [22,25,30,33]. To our knowledge, this is the first time that a study has shown the regulatory effect of glutamate on neuronostatin neurons.

These results revealed that the chemical signals of the peripheral injections of the agonists, as well as the antagonists, can reach to the neuronostatin neurons as we detected a significant neuronal activation (or blocking of the activation) following the injections. However, within the limitations of this study, it is not possible to suggest that this is a direct regulatory effect on the neuronostatin neurons. This could also be an indirect effect through the glutamate-receptive neurons located in the central nervous system.

The double immunochemistry technique we used in this study was limited to only the detectable levels of neuronostatin protein expressed by neurons and only these neurons were identified as neuronostatin neurons in the study. There may be other neuronostatin neurons in the central nervous system that we could not identify using this technique. A recent report showed that the somatostatin neurons localized in the tuberal nucleus regulated feeding [39], suggesting that these neurons could co-express neuronostatin. However, with immunohistochemistry, we, and others [2], could not detect neuronostatin immunoreactivity in the tuberal nucleus. 

Further studies using other methods, such as in situ hybridization, would be needed to understand the expression pattern of neuronostatin in hypothalamic areas other than the periventricular nucleus. We also did not analyze the expression levels of neuronostatin in individual neurons between fasting and refeeding groups, as the main aim of the study was to determine the rate of neuronal activation using double-labelling immunohistochemistry. Nevertheless, we determined that the number of neuronostatin-positive neurons in the periventricular nucleus did not differ between the fasting and refeeding groups.

In conclusion, our present study revealed that the glutamatergic system could stimulate the neuronostatin neurons through NMDA and non-NMDA glutamate receptors. Additionally, we demonstrated that a peripheral factor, such as refeeding, was effective in regulating the functions of neuronostatin neurons and that this effect was mediated through the glutamatergic system.
